# Knee strength ratios in competitive female athletes

**DOI:** 10.1371/journal.pone.0191077

**Published:** 2018-01-09

**Authors:** Jaroslaw Kabacinski, Michal Murawa, Krzysztof Mackala, Lechoslaw Bogdan Dworak

**Affiliations:** 1 Department of Biomechanics, Poznan University of Physical Education, Poznan, Poland; 2 Department of Track and Field, University School of Physical Education, Wroclaw, Poland; 3 Department of Bionics, University of the Arts, Poznan, Poland; University of Debrecen, HUNGARY

## Abstract

Knee strength ratios are related to the movement patterns, sport-specific training and knee injuries in athletes. The purpose of this study was to determine the ratios in the concentric isokinetic strength of the hamstrings and quadriceps and the isometric strength of the knee extensors. In female basketball players (*n* = 14) and female volleyball players (*n* = 12) were evaluated: the hamstrings to quadriceps peak torque ratio (H/Q) and side-to-side peak torque ratio (TR) for hamstrings and quadriceps; the ratio of the maximal bilateral strength to the summed maximal unilateral strength (B/U) and side-to-side maximal strength ratio (SR) for knee extensors. For the H/Q values, a 2 × 2 × 3 mixed-factorial analysis of variance and Bonferroni post hoc test were computed. The H/Q values increased from 48.0 (3.9)% at 60°/s to 70.4 (7.9)% at 300°/s. Furthermore, there were significant differences in the H/Q values between 300°/s and 180°/s, 300°/s and 60°/s in basketball and volleyball athletes, and between 180°/s and 60°/s only in basketball athletes (*p* < .05). Significantly higher H/Q results at 60°/s demonstrated basketball players than volleyball players (*p* < .05). Differences in the TR and SR mean values ranged from 4.4% to 8.6% and indicated no significant side-to-side strength deficits (*p* > .05). In both groups, greater isometric strength developed bilaterally was found (B/U > 100%). The findings revealed the magnitude of knee strength ratios in female athletes determined by sport-specific movements in basketball and volleyball. This study highlighted the importance of the bilateral strength deficit and muscular balance between the hamstrings and quadriceps in basketball and volleyball athletes in activities related to their movement patterns and specific training.

## Introduction

The magnitude of the hamstrings to quadriceps peak torque ratio (H/Q) may reflect the movement patterns during running or jumping [1–3]. A typical isokinetic concentric H/Q for healthy athletes ranges from 0.50 to 0.80 depending on angular velocity and indicates the muscle balance between the hamstrings and quadriceps [4,5]. Furthermore, the torque ratio of the agonist to antagonist knee muscles is associated with the demands of the sport, training adaptations and level of competition [1–3,6]. Previous studies evaluated the isokinetic H/Q in healthy volleyball and basketball players [1–4,7–11]. Several investigators have found an increased isokinetic H/Q with increased velocity in basketball athletes [1,7,11] and volleyball athletes [1,7]. Additionally, Rosene et al [1] noted no significant differences in the isokinetic H/Q between athletes in both sports.

Muscle strength is also compared between the non-dominant lower extremity (LE) and dominant LE [12]. In basketball and volleyball players, lateral dominance very often is determined by use of the left LE as the final foot before a take-off during jump shot in basketball and during spike in volleyball. Despite this, few authors have demonstrated no significant muscle strength differences between the non-dominant LE and dominant LE in basketball athletes [1,3,8,11] and volleyball athletes [1–3] The side-to-side muscle strength differences within the normative data (less than 10–15%) may emphasize the bilateral pattern of the specific activities of a sport [1–3,12].

An imbalance in the H/Q as well as the significant differences in muscle strength between the LEs are important risk factors of injury [4,13]. Therefore, thigh muscle strengthening and H/Q balancing may contribute to reduce the incidence of knee injuries in athletes [3,14–16]. Strong hamstrings may have a protective effect against excessive strain on the anterior cruciate ligament (ACL) in female athletes [14,17,18], whereas the increase of quadriceps strength (e.g., in eccentric training) can effectively prevent the development of patellar tendinopathy [18,19]. In particular, strength training program consisting of eccentric exercises is considered an effective strategy for preventing injuries related to jump landing [15,17,18].

Isometric and isokinetic tests can also be used to evaluate magnitude of the ratio of the strength developed during bilateral contraction and the sum of the strength developed by each limb independently (B/U). Especially, the isometric test is very useful to measure the B/U, because it minimizes extraneous factors such as change in muscle length and velocity of the movement [20]. Some investigations presented the isometric B/U and the isokinetic B/U of the knee extensors in untrained subjects [21–24]. Other studies focused on the B/U evaluation in weightlifters and cyclists [25] and in rowers with different levels of competition [26].

A bilateral deficit occurs when the bilateral strength is lower than the summed unilateral strength. Several studies have demonstrated the bilateral deficit (i.e., B/U < 100%) in different populations [20,27], age groups (young, middle-aged, old adults) [23,24], muscle groups (lower vs. upper extremities) [28] and actions [21,29–31]. This deficit may result from a reduced motor unit stimulation and reduced capacity to recruit fast-twitch fibers [30,32]. Another presumable cause of bilateral deficit includes the mechanism of motor neuron activation inhibition one limb by sensory activity from the contralateral limb and vice versa [22].

The purpose of the study was to determine the ratios in the concentric isokinetic torque of the hamstrings and quadriceps and the isometric muscle strength of the knee extensors developed in closed kinetic chain in female basketball and volleyball players. It was hypothesized that female volleyball players will present with a greater H/Q due to more specific action (spiking skills from 2 or 3 step approach and jumping to the block) occurring in the training and game than all specific movement occurring in basketball.

## Materials and methods

### Ethics statement

The study received approval from the Bioethical Committee of the Poznan University of Medical Sciences. All females provided written informed consent to participate in this research. All procedures were conducted according to the Declaration of Helsinki. The individual in this manuscript has given written informed consent (as outlined in PloS consent form) to publish these case details.

### Participants

Fourteen female basketball players and twelve female volleyball players participated in the study. Descriptive data for each group are shown in Table 1. All athletes were currently healthy and active with a Polish University Sports Association teams from the Polish first national leagues. Basketball players have been included in a special training program that incorporates numerous plyometric exercises during the preparatory phase. Volleyball players have been subjected to such exercises to a lesser degree. All female athletes exhibited left LE dominance. The dominant LE was defined as the limb with which the player performs take-off during jump.

**Table 1:** Mean (SD) values for participants’ characteristics.

Characteristic	Basketball athletes (*n* = 14)	Volleyball athletes (*n* = 12)
Age [years]	19.8 (1.4)	22.3 (4.2)
Body height [cm]	178.1 (7.7)	183.0 (8.7)
Body mass [kg]	67.6 (9.3)	74.4 (10.9)
BMI [kg/m^2^]	21.8 (2.1)	22.1 (1.8)

**Note:** BMI = body mass index; SD = standard deviation.

### Experimental procedures

Laboratory tests were performed in two independent sessions during the first round of the regular season (the second half of October). The 6-day period (session I) included the measurements of the concentric isokinetic torque of the hamstrings and quadriceps. During the 2-day period (session II), the isometric muscle strength of the knee extensors (KE) developed in closed kinetic chain (CKC) was examined. The following variables were evaluated: (a) the hamstrings to quadriceps peak torque ratio (H/Q) during unilateral contraction (isokinetic, formula: $\frac{H}{Q}\cdot 100\%$), (b) side-to-side peak torque ratio (TR) for hamstrings and quadriceps (isokinetic), (c) side-to-side maximal strength ratio (SR) for KE during unilateral contraction (isometric), SR and TR values were calculated using the formula: $\frac{X_{1}}{X_{2}}\cdot 100\%$, (where, X_**1**_<X_**2**_ and X_**1**_, X_**2**_ –strength of left LE or right LE, respectively), (d) the ratio of the maximal strength produced bilaterally to the sum of the maximal strength produced unilaterally (B/U) in CKC (isometric, formula: $\frac{B}{U}\cdot 100\%$).

The participants were instructed regarding the procedures of isokinetic and isometric tests and participants performed several repetition for familiarization. Prior to the tests, each female athlete performed ten minutes of a total body warm-up by on running on the treadmill and cycling on the stationary bike (Monark Ergomedic 874E) followed by five minutes of muscle stretching.

**Fig 1:**
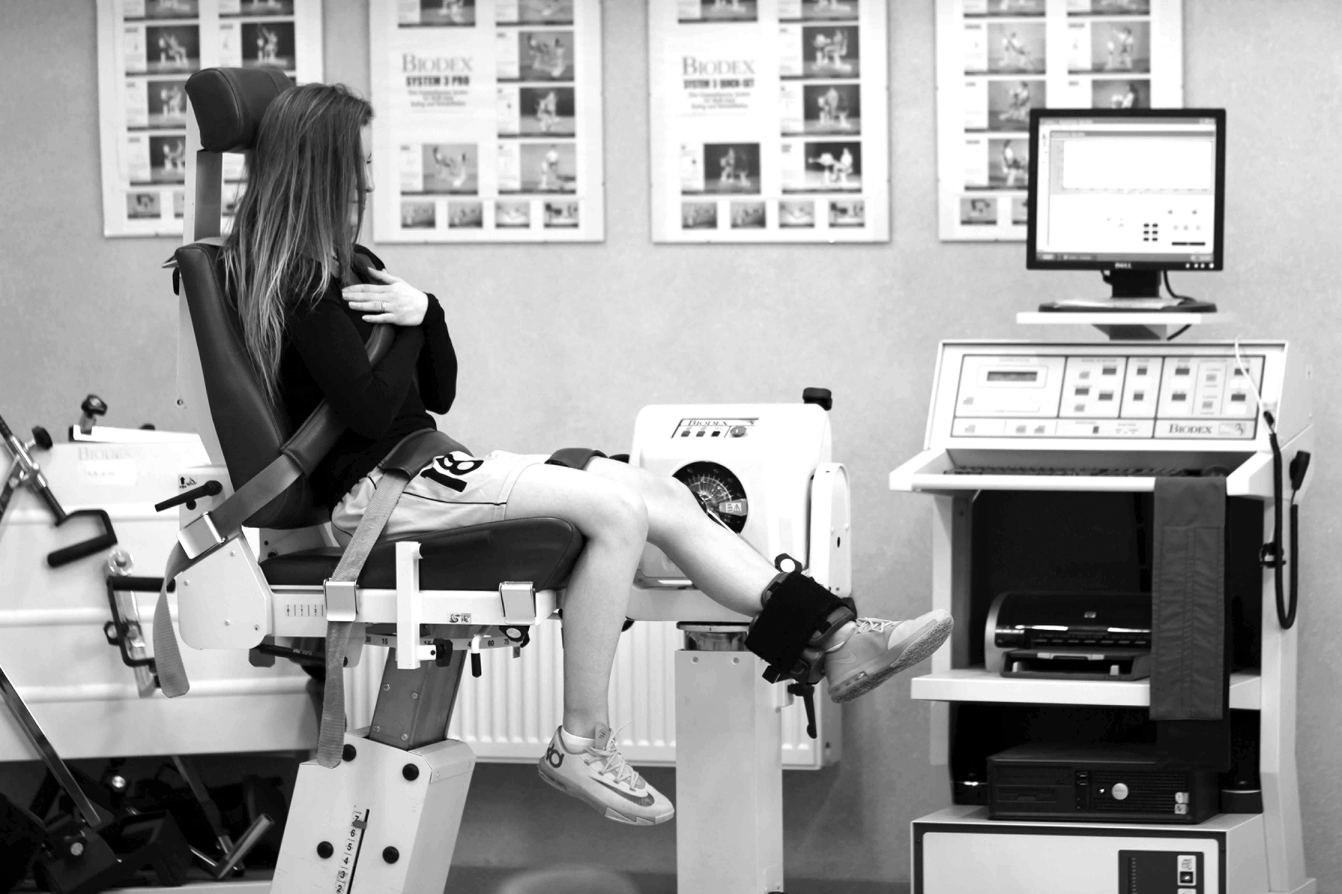
Test of the concentric isokinetic quadriceps and hamstrings torque on the Biodex 3 System.

Using the Biodex System 3 device (Biodex Medical Systems, Inc., Shirley, NY, USA) (Fig 1) we conducted the isokinetic test of concentric hamstrings and quadriceps strength at the following angular velocities: 60°/s, 180°/s and 300°/s. The following testing protocol was used: isokinetic bilateral, pattern: extension/flexion, mode: isokinetic, contraction: concentric, 4 series with angular velocities of 240°/s (7 trial repetitions as a warm-up), 300°/s (10 repetitions), 180°/s (7 repetitions) and 60°/s (5 repetitions) both for extension and flexion. During testing, the participant sat on an optimally positioned Biodex chair with stabilization straps at the trunk, hips and thigh while holding their arms across their chest. The knee joint rotation axis coincided with the rotation axis of the dynamometer. Gravity correction was made at both lower extremities at an incomplete knee extension position, so that valid data were obtained. Females performed maximal concentric knee flexion and extension of their dominant and non-dominant LEs, respectively, in the angular range of motion from 90° (flexion) to 0° (extension). Between series, a rest break of 60 seconds was given. Dynamometers calibration was performed before every session and in accordance with the manufacturer’s instructions.

**Fig 2:**
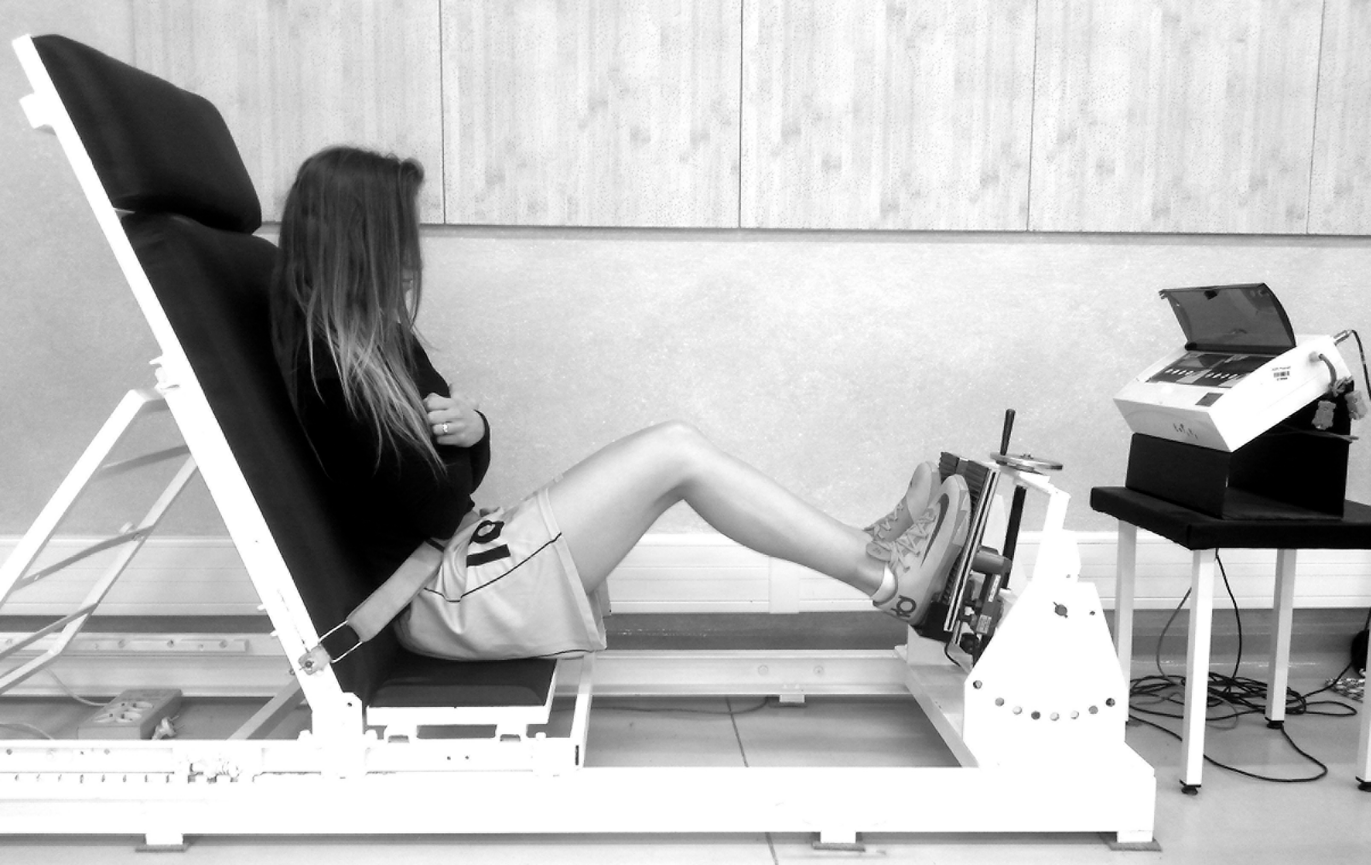
Test of the maximal isometric strength on the Strength Measuring Station-1 device in closed kinetic chain.

The isometric test was performed on the Strength Measuring Station-1 (SMS-1) device, equipped with the strain gauge dynamometer Scaime SB30X (measurement error ± 0.017%) and the indicator PUE 1 (RADWAG Company, Radom, Poland) (Fig 2). During the measurement, the participant sat with her hands on her chest, and her pelvis was blocked by a stabilizing belt. Hip, knee and ankle joints angles were 75°, 135° and 75°, respectively. The backrest of the seat and foot pedals was inclined at an 75° angle. Each female athlete pressed her foot on the pedals producing the maximal isometric strength of the KE, bilaterally (left and right LEs simultaneously) and unilaterally (left and right LEs independently). For each type of effort three maximal voluntary contractions (MVC) for 5 seconds were requested with an interval of 30 seconds between each MVC.

### Statistical analyses

Statistical analyses were performed in SPSS software for Windows (version 24.0; IBM Corp, Armonk, NY, USA). Mean and standard deviation (SD) of the data were calculated. Data were determined as normally distributed via the Shapiro-Wilk test. The differences in the values of maximal knee strength between the basketball and volleyball players, and between the left and right LEs were evaluated using the independent samples *t*-test and the paired samples *t*-test, respectively. For the H/Q values, a 2 × 2 × 3 (LE [left or right] × sport [basketball or volleyball] × velocity [60°/s, 180°/s or 300°/s]) mixed-factorial analysis of variance (ANOVA) was computed. Furthermore, Bonferroni post hoc test were used to determine differences in H/Q values between 60°/s, 180°/s and 300°/s. Sphericity assumption was verified by the Mauchly test. The level of statistical significance was set at *p* < .05.

## Results

**Fig 3:**
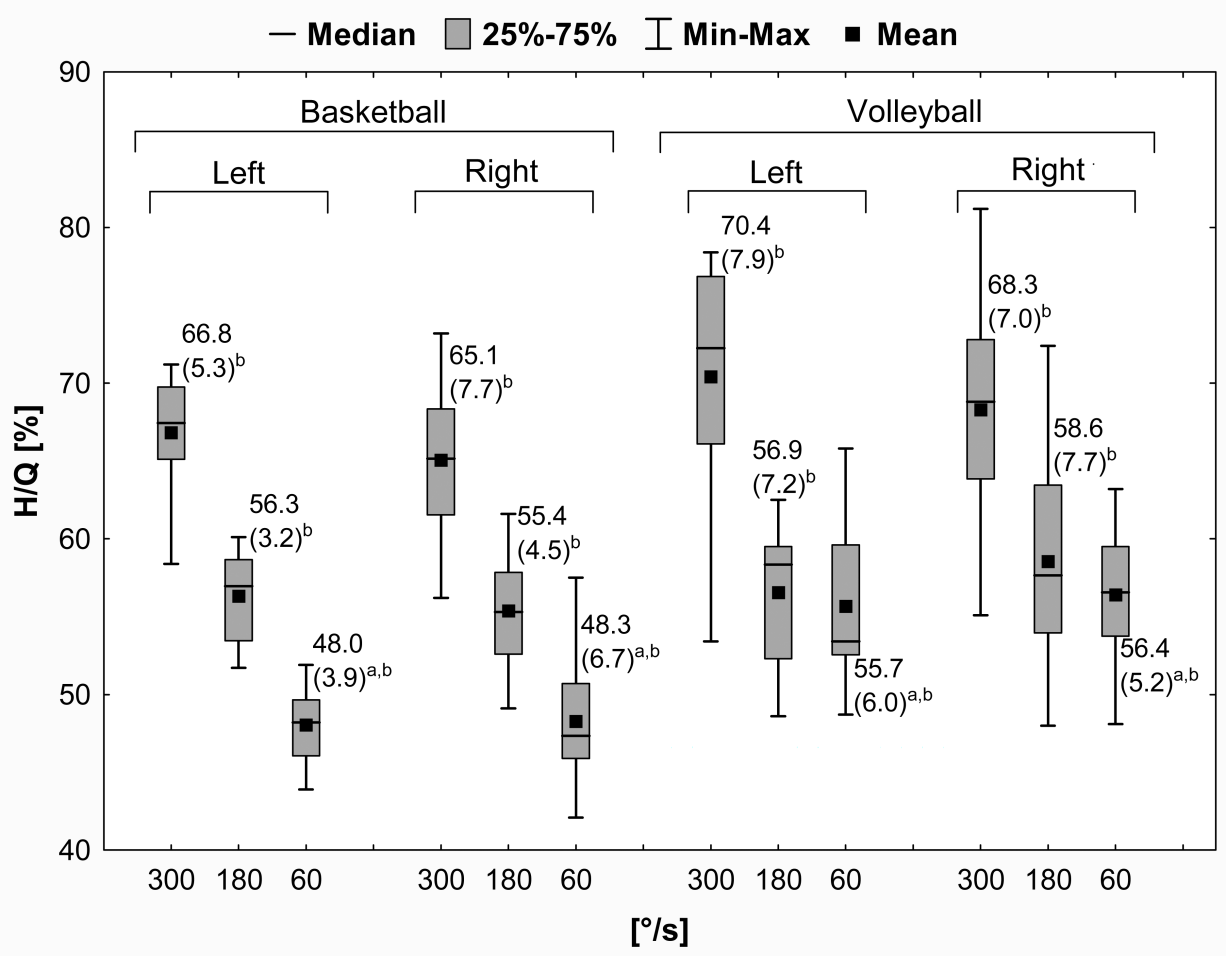
Mean (SD) values of the H/Q in female athletes. H/Q = hamstrings to quadriceps peak torque ratio; SD = standard deviation, 25% = first quartile; 75% = third quartile; min = minimum; max = maximum. ^a^ Significant differences (*p* < .05) in the H/Q values between the basketball and volleyball players; ^b^ Significant differences (*p* < .05) in the H/Q values between 60°/s and 180°/s, 180°/s and 300°/s, and 60°/s and 300°/s.

The H/Q results in female basketball and volleyball players are presented in Fig 3. The H/Q values decreased with the decrease in velocity from 66.8 (5.3)% (300°/s) to 48.0 (3.9)% (60°/s) in basketball players and 70.4 (7.9)% (300°/s) to 55.7 (6.0)% (60°/s) in volleyball players. Furthermore, were found significant differences in the H/Q values between the 60°/s, 180°/s and 300°/s and between the basketball and volleyball players for sport × velocity factors (ANOVA test, *p* < .05). Using the Mauchly test, was provided the sphericity assumption at level *W* = 0.93 and *p* = 0.21.

The H/Q values were significantly higher (Bonferroni post hoc test, *p* < .05): (1) for 300°/s than for 180°/s, about 10.5% (left LE) and 8.7% (right LE) in basketball athletes, and about 13.5% (left LE) and 9.7% (right LE) in volleyball athletes, (2) for 300°/s than for 60°/s, about 18.8% (left LE) and 16.8% (right LE) in basketball athletes, and about 14.7% (left LE) and 11.9% (right LE) in volleyball athletes, (3) for 180°/s than for 60°/s, about 8.3% (left LE) and 7.1% (right LE) in basketball players. In the case of 60°/s velocity, were noted significantly greater the H/Q values (*p* < .05) in volleyball athletes compared to the basketball athletes, about 13.8% (left LE) and 14.4% (right LE). Moreover, no significant differences in the H/Q between the left and right LEs were demonstrated (*p* > .05).

**Fig 4:**
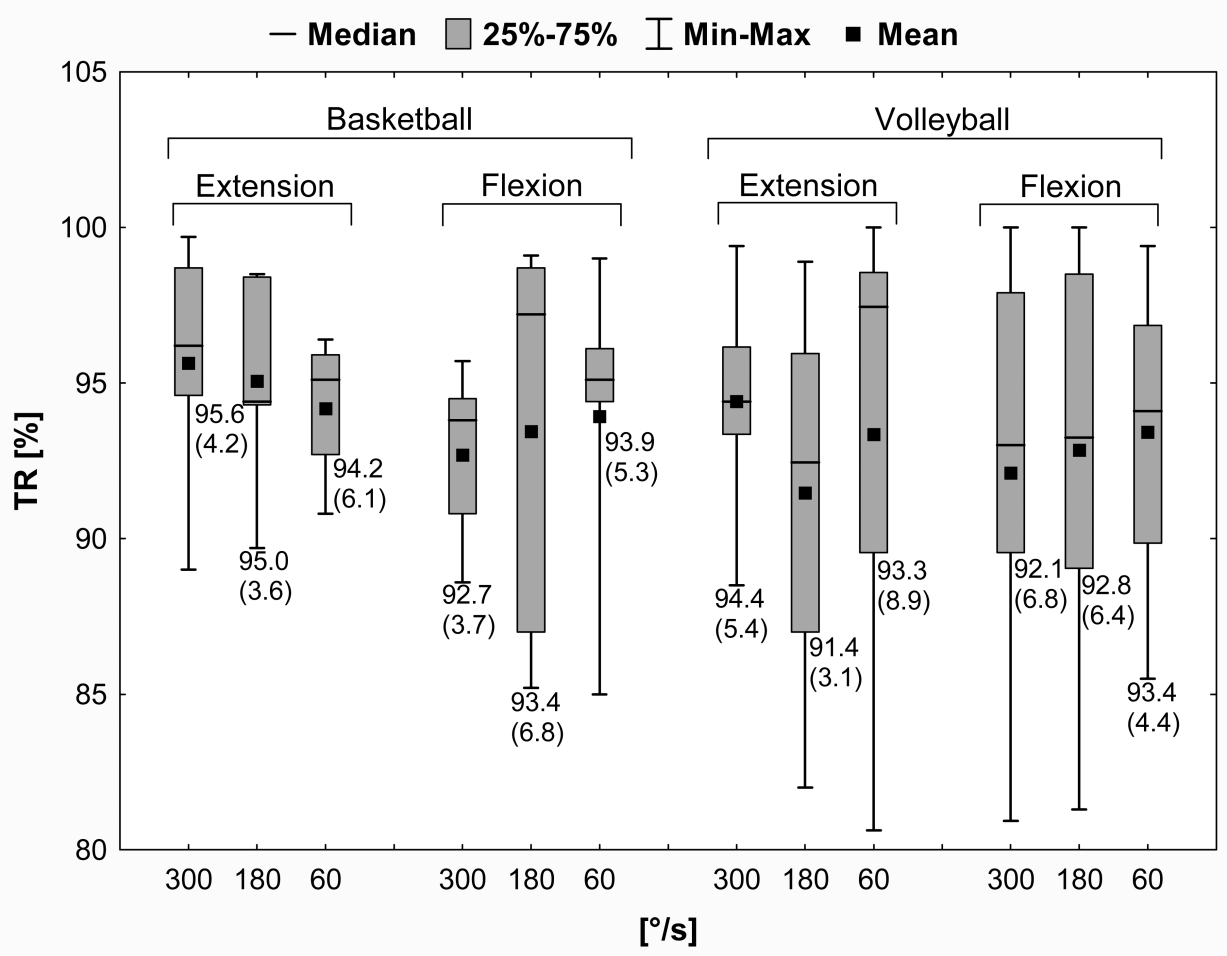
Mean (SD) values of the TR for quadriceps and hamstrings in female athletes. TR = side-to-side peak torque ratio for quadriceps and hamstrings; SD = standard deviation; 25% = first quartile; 75% = third quartile; min = minimum; max = maximum.

Statistical analysis showed no significant differences in the quadriceps and hamstrings peak torque values between the left and right LEs (the paired samples *t*-test, *p* > .05) and no significant TR values between the basketball and volleyball players (the independent samples *t*-test, *p* > .05). Differences in the TR mean values were below 10% (no significant strength deficit) and ranged from 4.4% to 7.3% in basketball players and from 5.6% to 8.6% in volleyball players. The results of the TR are presented in Fig 4.

**Fig 5:**
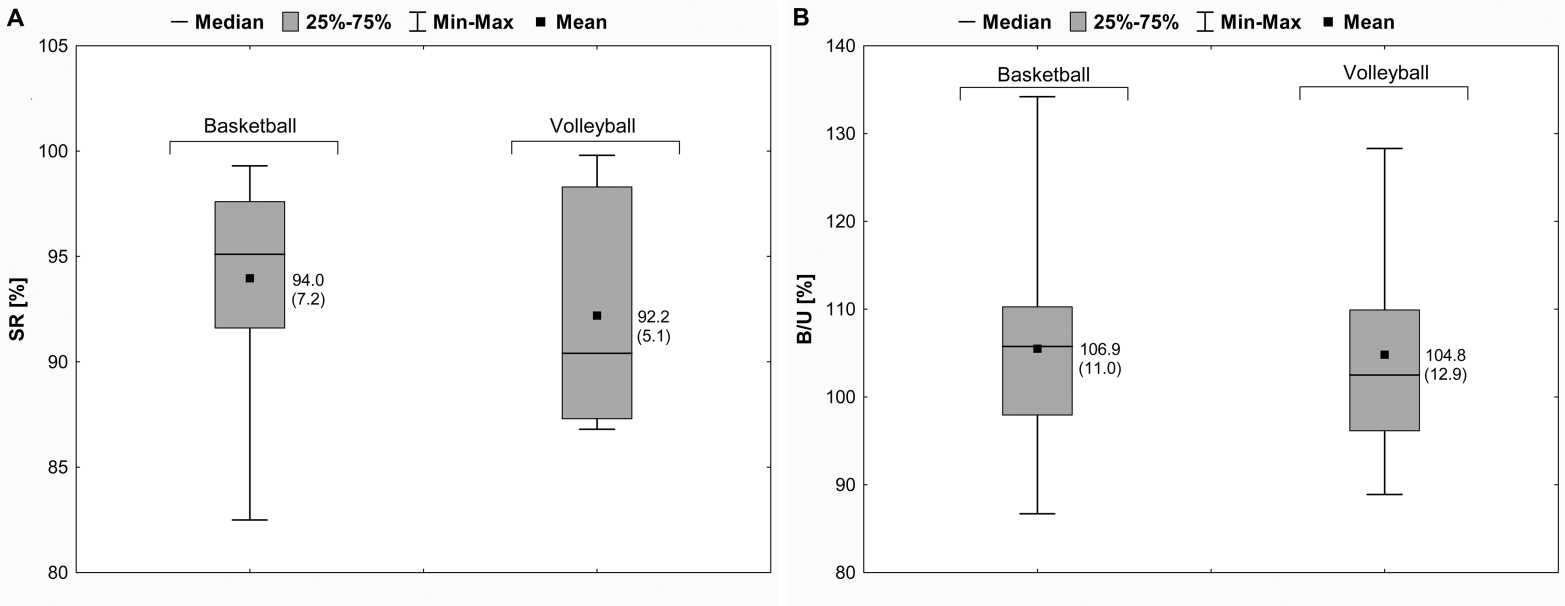
Mean (SD) values of the isometric strength ratios for KE in female athletes. (A) SR = side-to-side maximal strength ratio for KE; (B) B/U = ratio of the maximal strength produced bilaterally to the sum of the maximal strength produced unilaterally for KE. KE = knee extensors; SD = standard deviation; 25% = first quartile; 75% = third quartile; min = minimum; max = maximum.

In female athletes, there were no significant differences in the KE strength values between the left and right LEs (the paired samples *t*-test, *p* > .05). The SR results revealed no significant side-to-side strength deficits: 6.0% (basketball players) and 7.8% (volleyball players) (Fig 5A). The B/U values were greater than 100% about 6.9% in basketball athletes and about 4.8% in volleyball athletes (Fig 5B). Moreover, no significant differences in the SR and B/U values between the basketball and volleyball players were found (the independent samples *t*-test, *p* > .05).

## Discussion

The present study evaluated the concentric H/Q of female volleyball and basketball athletes. The findings revealed the knee muscle strength balance (H/Q ranged of approximately 50–70%) at 60°/s, 180°/s and 300°/s velocities. The H/Q results from 50% to 80%–typical for healthy people–are recommended for normal knee joint function [4,5]. The magnitude of this ratio can be determined by sport-specific demands related to movement patterns during running or jumping [1–3]. Furthermore, reduced function of the hamstrings due to activities that emphasize loads on the knee extensors may lead to the muscle strength imbalance between the hamstrings and quadriceps which is implicated as a potential risk factor of knee injuries [1,13]. Increased dynamic loads during jumping (e.g., in volleyball and basketball), cutting and pivoting (in basketball) as well as muscular imbalance between the hamstrings and quadriceps may contribute to the ACL injury [4]. Therefore, some authors have indicated the additional exercises increasing hamstrings strength to protect against excessive ACL strain, especially in athletes with significantly low H/Q [14,17,18].

Significant differences in the H/Q values at 60°/s velocity between both groups were noted. Thus, quadriceps relative to hamstrings were significantly stronger among basketball athletes than volleyball athletes. The results from this study supported the hypothesis that female volleyball players show greater H/Q. It can be explained by more specific action occurring in volleyball, in particular during spiking where the hamstrings act eccentrically during 2 or 3 steps of approach (acceleration). During late swing, the hip is flexed and the knee is extending. The hamstring muscles are active at this stage while lengthening, which could induce an strong eccentric contraction, where into stance phase, hamstring muscles remain active presumably shortening which could induce a concentric contraction [33,34]. Studies of Mackala et al [35] and Coh and Mackala [36] have shown that the hip extensors (hamstrings) are very likely contributors of vertical jump height during take-off performance, where remain active eccentrically.

Isokinetic evaluation showed a reduction in the H/Q values with decrease in velocity due to the significant increase in peak torque values of quadriceps group. Knee flexors and knee extensors are responsible for different functions; e.g., during volleyball and basketball jumps, quadriceps group primarily counterbalance adverse external loads in landing phase. Furthermore, there were significant H/Q differences between the three angular velocities only in basketball players which obtained significantly higher H/Q values at 300°/s than at 180°/s and 60°/s as well as significantly higher values at 180°/s than 60°/s. Similarly, Rosene et al [1] noted significant increases in the H/Q results from 48.8% (left LE) and 50.1% (right LE) at 60°/s to 53.5% (left LE) and 56.4% (right LE) at 120°/s, and to 58.7% (left LE) and 59.4% (right LE) at 180°/s among intercollegiate female soccer, softball, volleyball and basketball athletes.

The H/Q values in athletes of various sports at similar competitive levels may depend on the sport-specific demands. For example, basketball players perform the rapid change of position (deceleration and acceleration) during running, pivoting and other jumping techniques compared to the volleyball players. This study demonstrated significantly higher the H/Q in volleyball players than basketball players. In turn, Cheung et al [3] showed significant differences in the H/Q values between field and court players (basketball and volleyball) at 60°/s for dominant LE and at 300°/s for non-dominant LE. In contrast, Rosene et al [1] found no significant differences in the H/Q between soccer, softball, volleyball and basketball athletes. Furthermore, Zakas [6] noted no significant differences of this ratio at 60°/s and 180°/s between basketball and soccer players.

Comparative analysis between limbs revealed no significant differences in muscle strength between the left and right LEs in female athletes of the both sports. Other studies also showed no significant side-to-side strength differences in volleyball players [1–3,37] and basketball players [1,3,6,8,11]. Considering women only, no significant peak torque differences between non-dominant and dominant LEs were found by Rosene et al [1] in female volleyball and basketball athletes and Rouis et al [11] in female basketball athletes. These non-significant strength deficits in athletes were explained through specific loads placed on the LEs to maintain similar strength on both sides. Both in the basketball and volleyball athletes, the LEs are subjected to bilateral loads during defensive and offensive techniques, which very often requires double-leg performance that reduces the side-to-side differences in muscle strength. Furthermore, no significant knee strength differences between the left and right LEs can emphasize bilateral pattern of some basketball and volleyball skills, nevertheless that athletes very often used the left LE as the final limb during take-off before a shot jump in basketball or before a spike jump in volleyball. Undoubtedly, the effective execution of these specific sport’s movements requires strength enhancement of preferred side.

Previous studies compared the isokinetic strength deficit between athletes of various sports. Rosene et al [1] reported that side-to-side thigh muscle strength was not significant between volleyball, soccer, basketball and softball athletes. Moreover, Cheung et al [3] found no significant differences in the normalized peak torque of hamstrings and quadriceps between athletes playing field (soccer) and court (volleyball and basketball) sports. This investigation also demonstrated no significant peak torque deficit between the basketball and volleyball players, which can determine similar bilateral movement patterns during jumping and landing. However, Magalhães et al [2] showed a significant greater the hamstrings strength difference between non-dominant and dominant LEs in soccer players versus volleyball players at 90°/s velocity. This pattern was explained by higher unilateral demands of the hamstrings muscles in stabilizing actions during kicking and passing of the ball in soccer [2,38].

The B/U results were comparable between the basketball and volleyball athletes and higher than 100%. The participants developed a greater strength of the KE bilaterally due to the better use of force capabilities both the LEs simultaneously. The bilateral strength greater than the sum of the unilateral strength (B/U > 100%) is defined as bilateral facilitation and may result from a neuromuscular adaptations of the KE to performing bilateral tasks. In basketball and volleyball athletes, bilateral loads of the LEs during cutting, jumping or landing may contribute to the bilateral facilitation.

The B/U below 100% indicates the bilateral deficit. Some investigators have suggested that reduced capacity to recruit fast-twitch fibers may cause this deficit [30,32]. Other authors indicated the neural factor, related to the reduction in the recruitment of motor units and consequent limb torque generation, resulted in a decreased linear relationship between torque and electrical muscle activity in the bilateral condition [21,23,29]. Furthermore, Botton et al [21] found a greater sum of the peak torque produced unilaterally compared with the peak torque produced bilaterally, possibly because of a neural inhibition that occurs during the bilateral condition, which inhibits maximal torque production.

The B/U results in athletes of selected sports depend on the level of competition, unilateral or bilateral exercises as well as movement patterns. For example, Sale [26] reported a small bilateral deficit that occurred in national rowers, whereas high performance rowers had greater muscle strength developed bilaterally. Fleck and Kreamer [25] found the B/U > 100% in athletes loading the LEs simultaneously (e.g., weightlifters) and the B/U < 100% in athletes performing alternating movement of the LEs (e.g., cyclists). In addition, Beurskens et al [22] showed a decreased bilateral deficit in young and old adults after bilateral heavy-resistance training and balance training. Furthermore, Botton et al [31] evaluated the B/U in recreationally active young women and demonstrated a significant bilateral deficit in subjects after unilateral training and significant bilateral facilitation in subjects after bilateral training.

## Conclusions

Thigh muscles strengthening, the bilateral strength deficit reduction and H/Q balancing are often considered important activities, both for high performance in sport and knee injuries prevention. A significantly greater H/Q at 60°/s in volleyball athletes indicates better utilization of hamstring muscle group in volleyball specific movements or a weakening of the quadriceps relative to the hamstrings compared with the basketball players. It should be emphasized that each LE needs to be used in any training environment, to improve performance and reduce injury. Furthermore, providing rational stimulus (muscle strength, plyometric or some speed activities) for both LEs can prevent any imbalances resulted from repetitive unilateral movements (strong dominance of preferred limb) of athlete’s specific sport movements. In addition, specific bilateral exercises of muscles LEs can increase the B/U. Although, bilateral deficit in some athletes remains without significant changes, despite the involvement of bilateral exercises [25]. In turn, the use of unilateral exercises is important for activities requiring increased strength developed by each limb independently. Thus, sport specific training and movements patterns determine the magnitude of the deficit bilateral as well as differences in strength between limbs and muscular balance between the hamstrings and quadriceps.
